# Dynamics Research of the Hopfield Neural Network Based on Hyperbolic Tangent Memristor with Absolute Value

**DOI:** 10.3390/mi16020228

**Published:** 2025-02-17

**Authors:** Huiyan Gao, Hongmei Xu

**Affiliations:** School of Engineering, Yanbian University, Yanji 133002, China; 2023050042@ybu.edu.cn

**Keywords:** absolute value hyperbolic tangent memristor, Hopfield neural network, coexistence attractor, FPGA

## Abstract

Neurons in the brain are interconnected through synapses. Local active memristors can both simulate the synaptic behavior of neurons and the action potentials of neurons. Currently, the hyperbolic tangent function-type memristors used for coupling neural networks do not belong to local active memristors. To take advantage of local active memristors and consider the multi-equilibrium point problem, a cosine function is introduced into the state equation, resulting in the design of an absolute value hyperbolic tangent-type double local active memristor, and it is used as a coupling synapse to replace a synaptic weight in a 3-neuron HNN. Then, basic dynamical analysis methods are used to study the effects of different memristor synapse coupling strengths and different initial conditions on the dynamics of the neural network. The research results indicate that dynamical behavior of memristor Hopfield neural network is closely related to the synaptic coupling strengths and the initial conditions, and this neural network exhibits rich dynamical behaviors, including the coexistence of chaotic and periodic attractors, super-multistability phenomena, etc. Finally, the neural network was implemented using an FPGA development board, verifying the hardware feasibility of this system.

## 1. Introduction

The human brain is composed of billions of neurons, which are interconnected through synapses, forming a highly complex neural network [1]. Therefore, constructing artificial neural network models is an important foundation for studying the human brain. In 1984, Professor J.J. Hopfield proposed a fully connected neural network model based on biological neural systems: the Hopfield neural network [2], also known as HNN. This neural network model can produce some simple dynamic behaviors [3,4,5,6,7], but traditional neural network models use fixed resistors to simulate the synaptic strength between neurons, resulting in unchangeable synaptic strength, which leads to limitations and weak nonlinearity, making it impossible to simulate the chaotic characteristics of biological neural systems. However, the memristor’s memconductance is related to its own voltage or current, which makes its resistance value variable, thereby allowing synaptic strength to be variable, enabling a more effective simulation of the chaotic characteristics of biological neural systems [8,9,10]. Therefore, many scholars have introduced memristors when constructing artificial neural network models. In 2023, Sun Liang and others [11] proposed an improved multi-stable memristor, replacing a synaptic weight in a 4-neuron HNN. They studied the dynamic characteristics related to the memristor synapse coupling strengths and designed a chaotic image encryption system. In 2023, Huang Lili and others [12] proposed an HNN model with absolute value memristor-coupled synapse strengths, studying the coexistence behavior of symmetric attractors under different initial values. In 2019, Chen Chengjie and others [13], using an ideal memristor to simulate electromagnetic induction in a dual-neuron HNN, studied the dynamical behavior of the system under varying coupling strengths and the coexistence bifurcation under different initial values. From the above research, it can be concluded that memristors can effectively simulate the strength of synaptic connections between neurons in neural networks due to their diverse significant characteristics [14], and these neural network models that memristor-coupled have synaptic weight variability, which can make the dynamic behavior of the neural network more complex, providing support for studying the chaotic characteristics of biological neural systems. Precisely because of this, memristor-coupled neural networks have great potential and advantages in both technology and computational protocols for artificial intelligence [15].

Memristors can be divided into local passive memristors and local active memristors. Local passive memristors can be used to simulate synaptic behavior, while local active memristors can simulate neuronal functions. Local active memristors have the ability to generate oscillations and amplify weak signals in nonlinear dynamical systems [16]. When applied in neural network models, they can easily induce various types of neuro-morphic discharge behaviors. Therefore, using local active memristors to simulate biological synapses when constructing artificial neural networks can make the dynamical systems more complex [17]. Currently, many scholars have proposed Hopfield neural networks coupled with local active memristors. In 2020, Lin Hairong and others [18] proposed a local active memristor model with hyper-multiple steady states, replacing one synaptic weight in a 4-neuron HNN. They studied the impact of synaptic coupling strength on dynamic behavior and observed the phenomenon of infinity many coexisting chaotic attractors. In 2022, Li Chunlai and others [19] proposed a three-state local active memristor model, replacing one synaptic weight in a 3-neuron HNN. The study found that the distribution of the system’s equilibrium points depends on the coupling weight of the memristive synapses. In 2024, Wang Mengjiao and others [20] proposed a composite exponential local active memristor model, replacing a synaptic weight in a 4-neuron HNN. They studied the system’s dynamic behavior under variations in the memristor’s internal parameters and the frequency of external stimulus currents and discovered a chaotic state over a wide range of parameters.

Although many Hopfield neural networks with local active memristor coupling have been proposed, the mathematical model of local active memristors with hyperbolic tangent function coupling to HNN has not yet been introduced. The hyperbolic tangent function exhibits smooth saturation and S-shaped nonlinear characteristics; its S-shaped curve closely resembles the activation functions commonly used in neuromorphic computing, making it a natural fit for memristive systems. Moreover, compared to other nonlinear functions, such as sigmoid or polynomial functions, the hyperbolic tangent function provides both mathematical simplicity and physical interpretability, ensuring stable and continuous memristive behavior. The most important thing is that it is known that the hyperbolic tangent function tanh(*x*) has good nonlinear characteristics [21]. Considering that local active regions are the origin of complexity, neural networks coupled with locally active memristors can produce richer dynamic behaviors [22] and the hyperbolic tangent function can generate local active intervals through simple transformations. Therefore, we propose a hyperbolic tangent-type memristor. In this paper, the independent variable *x* in the hyperbolic tangent function is taken as its absolute value, and, on this basis, an adjustable parameter *n* is added to the independent variable so that this memristor can generate a local active region, and the local active region changes with the variation in parameter *n*. Moreover, due to the presence of multiple equilibrium points in local active memristors, the system exhibits multistability and complexity [23]. Therefore, when designing the memristor model, a cosine function is added to the state equation.

Referring to the above discussion, this paper proposes an absolute value hyperbolic tangent memristor, replacing one synaptic weight in a 3-neuron HNN. Using basic dynamical analysis methods, the influence of different memristor synaptic coupling strengths and different initial conditions on the dynamics of the neural network was studied, revealing that this neural network exhibits multistability phenomena. Finally, the system was implemented on an FPGA platform.

## 2. Dual Local Active Memristor Model

### 2.1. Mathematical Model of Memristor

The mathematical model of the voltage-controlled memristor is as follows:

(1) $$\left\{\begin{array}{c} \;\;i=G\left(x\right)v\;\;\;\\ \;\;\frac{dx}{dt}=g\left(x,v\right) \end{array}\right.$$

Here, *i* represents the current applied across the memristor, which is equal to the product of the memristor’s memconductance $G\left(x\right)$ and voltage v accross the memristor, and dx/dt represents the rate of change in the internal state variable x of the memristor. Due to the close relationship between local active regions and biological synapses, and compared to passive memristors, local active memristors with multiple equilibrium points can generate more complex dynamic behaviors. Therefore, based on the above equation, a cosine function is introduced into the internal state function of the memristor, leading to the proposal of a new mathematical model for the dual local active memristor:

(2) $$\left\{\;\;\begin{array}{c} i=\tanh\left(\left|x\right|+n\right)v\\ \frac{dx}{dt}=\cos\left(x\right)+v\;\;\;\;\;\; \end{array}\right.$$

**Figure 1:**
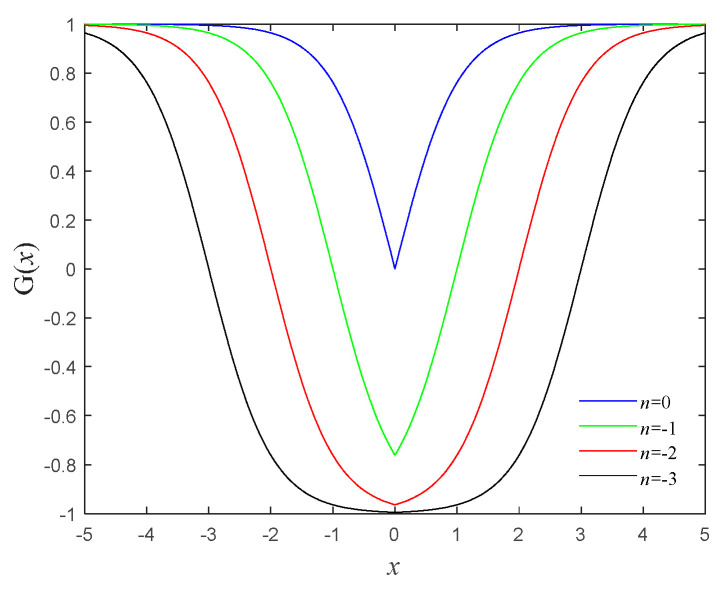
The relationship between $G\left(x\right)$ and n.

Here, memconductance $G\left(x\right)=\tan h\left(\left|x\right|+n\right)$, *n* is an internal parameter of the memristor. The relationship between the memconductance $G\left(x\right)$ and the internal parameter n is shown in Figure 1.

From Figure 1, it can be seen that when n<0, the interval where $G\left(x\right)<0$ begins to appear. As the value of n gradually decreases, the area where $G\left(x\right)<0$ gradually increases. Therefore, when n<0, there must exist an interval $G\left(x\right)<0$ in which this memristor is a local active memristor. Hence, the internal parameters are determined as n=−3, and Formula (2) should be rewritten as the following:

(3) $$\left\{\;\;\begin{array}{c} i=\tanh\left(\left|x\right|-3\right)v\\ \frac{dx}{dt}=\cos\left(x\right)+v\;\;\;\;\;\; \end{array}\right.$$

According to Kirchhoff’s laws, the circuit equation for the memristor can be expressed as follows:

(4) $$\left\{\begin{array}{c} i=G\left(v_{z}\right)v=\frac{1}{R_{L}}\tanh\left(\left|v_{z}\right|-3\right)v\\ RC\frac{dv_{z}}{dt}=\cos\left(v_{z}\right)+v \end{array}\right.$$

**Figure 2:**
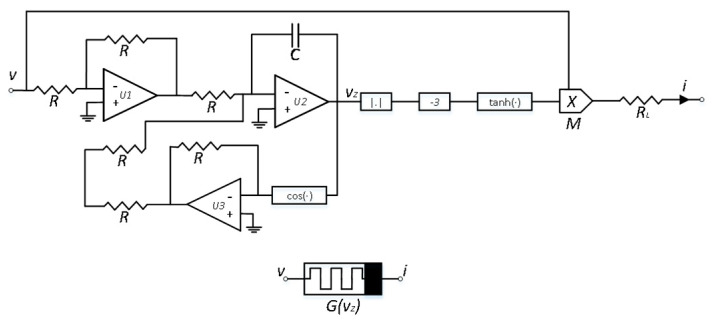
Memristor circuit.

In the above equation, the memristor state x is simulated by the output voltage of integrator, namely $v_{z}$. Based on Equation (4), its equivalent circuit can be implemented, as shown in Figure 2. Among them, U is the analog operational amplifier, M is the analog multiplier. The hyperbolic tangent function and the absolute value function are both implemented using the hyperbolic tangent circuit and the absolute value circuit proposed in reference [24].

### 2.2. Hysteresis Loop and Time Domain Waveform

**Figure 3:**
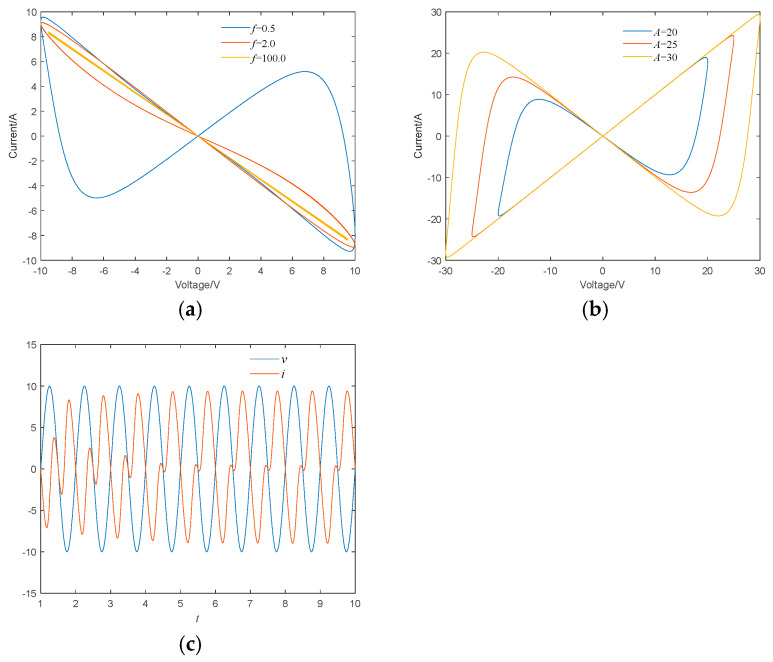
Hysteresis loops and time domain waveform of memristor: (**a**) hysteresis loops at different frequencies; (**b**) hysteresis loops at different amplitudes; and (**c**) time domain waveforms of voltage and current.

When the input signal across the memristor was set as $v=Asin\left(2\pi ft\right)$, the hysteresis loops of the proposed memristor under different frequencies and amplitudes were studied. When $A=10V,\;x_{0}=1$, the input signal frequencies are set to 0.5, 2.0, and 100.0, respectively, and the hysteresis loop of the memristor is shown in Figure 3a. As shown in Figure 3a, with the gradual increase in frequency, the side lobe area of the hysteresis loop gradually decreases, which is consistent with the frequency change effect of the memristor. Furthermore, when the frequency is high enough, the hysteresis loop compresses into a straight line. When $f=1\mathrm{Hz},x_{0}=1$, the input signal amplitudes are set to 20, 25, and 30, and the hysteresis loop of the memristor is shown in Figure 3b. As shown in Figure 3b, with the gradual increase in amplitude, the side lobe area of the pinched hysteresis loop also gradually increases, which is consistent with the fingerprint characteristics of the memristor. The hysteresis loop of a memristor not only reflects nonlinearity but also demonstrates its memory characteristics. The nonlinear and memory characteristics of memristors enable them to simulate the synaptic plasticity of biological neurons. In neural networks, the changes in synaptic weights are the basis of learning and memory, and the hysteresis loop behavior of memristors can naturally achieve this weight change. The nonlinear behavior of memristors which is exhibited in the hysteresis loop can introduce more complex dynamic characteristics, such as multistability and chaotic behavior, which helps improve the performance of neural networks in complex tasks. For example, in the practical application of image encryption, memristor-based chaotic systems can provide higher security. When $A=10\;V,\;f=1\;\mathrm{Hz},\;x_{0}=1$, the time domain waveforms of the voltage and current across the resistor are shown in Figure 3c. As shown in Figure 3c, when the voltage v across the memristor is 0, the current i through the memristor is also 0; however, when the current i through the memristor is 0, the voltage v across the memristor may not necessarily be 0, which is consistent with the memristor’s consistent zero-crossing and inconsistent zero-crossing characteristics [25].

### 2.3. Power-Off Plot and DC Voltage-Current

To determine whether a memristor is a non-volatile memristor, one needs to look for answers in the Power-off plot (POP) of the change rate dx/dt. When dx/dt=0, the POP of a memristor has two or more points with a negative slope, indicating it is a non-volatile memristor [26]. Therefore, substituting v=0 into the state Equation (3), we can obtain the following:

(5) $$\frac{dx}{dt}=g\left(x,0\right)=\cos\left(x\right)$$

**Figure 4:**
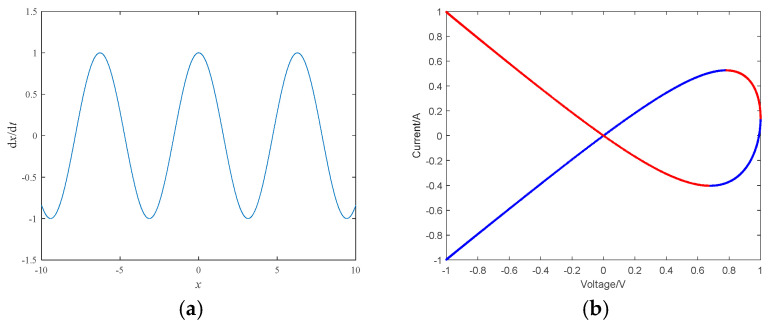
POP and DC V-I diagram of memristor: (**a**) POP; (**b**) DC V-I diagram.

The Power-off plot within the interval $x\in \left(-10,10\right)$ is shown in Figure 4a. It is known that each intersection point of the POP with the horizontal axis is a balance point of the memristor, hence the memristor has an infinite number of equilibrium points. As shown in Figure 4a, the POP has three intersections with negative slopes within the interval $\left(-10,10\right)$, which is the characteristic of non-volatile memristors.

To determine whether a memristor is a dual local active memristor, one must look for answers in the DC voltage-current (DC V-I). If the DC voltage-current curve contains two intervals with negative slopes, it indicates that there are two local active intervals within the memristor. Therefore, let dx/dt=0, we can obtain $V=-\cos\left(X\right)$, where *V* represents the DC voltage, and *X* represents the equilibrium point where dx/dt=0. Substituting it into Equation (3), we can obtain the relationship between the direct current *I* and the direct voltage *V* as the following:

(6) $$I=-\cos\left(X\right)\tan h\left(\left|X\right|-3\right)$$

The DC V-I curve within the interval $X\in \left(0,2\pi \right)$ is shown in Figure 4b where the slope of the blue curve is positive and the slope of the red curve is negative. From Figure 4b, it can be seen that when −1<X<0.6691 and 0.7826<X<1, d*I*/d*V* < 0. Therefore, this memristor has two local active intervals, which is the characteristic of a dual local active memristor [27].

## 3. Modeling of Memristor-Coupled Three-Neuron HNN

The Hopfield neural network model typically consists of n neurons, which can be represented as the following [28]:

(7) $$C_{i}\frac{dx_{i}}{dt}=-\frac{x_{i}}{R_{i}}+\sum_{j=1}^{n}w_{ij}\tan h\left(x_{j}\right)+I_{i}$$

Among them, $C_{i}$, $R_{i}$, and $x_{i}$ are the capacitance, resistance, and membrane voltage of the i-th neuron, respectively. $w_{ij}$ is the synaptic weight of the connection strength from the j-th neuron to the i-th neuron, that is, the amount of influence one neuron’s firing has on another neuron; $\tan h\left(\cdot \right)$ represents the neuron activation function, and $I_{i}$ represents the external input current.

**Figure 5:**
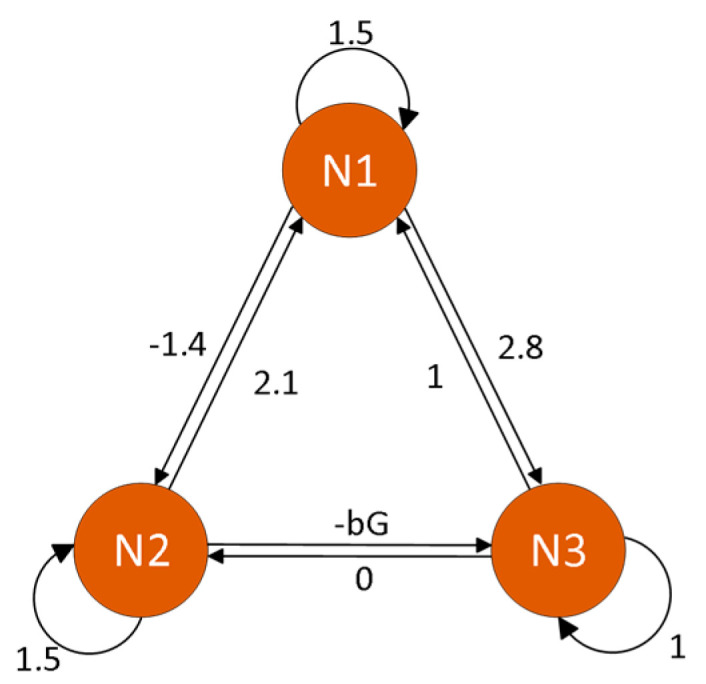
Topological structure diagram of memristor coupled HNN.

According to memristor theory, memcomductance $G\left(x\right)$ and synaptic weight $w_{ij}$ are both dimensions of conductance [29]. Therefore, the synaptic weight between neuron 2 and neuron 3 in the 3-neuron HNN can be replaced with the memconductance $G\left(x\right)$, that is, by setting $w_{32}=-bG\left(x\right)$, the topology of the 3-neuron Hopfield neural network is shown in Figure 5.

Let $R_{i}=1$, $C_{i}=1$, and the external stimulus currents $I_{1}=I_{2}=I_{3}=I_{4}=0$. Substituting these values into the proposed dual local active memristor mathematical model, and through extensive numerical simulations, the synaptic weight matrix of this system was obtained as follows:

(8) $$W=\left[\begin{array}{c} w_{11}\;\;\;\;\;w_{12}\;\;\;\;\;w_{13}\\ w_{21}\;\;\;\;\;w_{22}\;\;\;\;\;w_{23}\\ w_{31}\;\;\;\;\;w_{32}\;\;\;\;\;w_{33} \end{array}\right]=\left[\begin{array}{c} \;\;1.5\;\;\;\;\;\;\;\;\;2.1\;\;\;\;\;\;\;1\\ -1.4\;\;\;\;\;\;\;\;1.5\;\;\;\;\;\;\;0\;\\ \;\;2.8\;\;\;-\mathrm{bG}\;\;\;\;\;\;\;1 \end{array}\right]\;$$

Based on the above matrix, the absolute value hyperbolic tangent-type dual local active memristor-coupled Hopfield neural network model proposed in this paper can be obtained as follows:

(9) $$\left\{\begin{array}{l} \dot{x_{1}}=-x_{1}+1.5\tanh\left(x_{1}\right)+2.1\tanh\left(x_{2}\right)+\tanh\left(x_{3}\right)\\ \dot{x_{2}}=-x_{2}-1.4\tanh\left(x_{1}\right)+1.5\tanh\left(x_{2}\right)\\ \dot{x_{3}}=-x_{3}+2.8\tanh\left(x_{1}\right)-b\tanh\left(\left|z\right|-3\right)\tanh\left(x_{2}\right)+\tanh\left(x_{3}\right)\\ z=\cos\left(z\right)+\tanh\left(x_{2}\right) \end{array}\right.$$

## 4. Dynamic Analysis of the Memoristor-Coupled Three-Neuron HNN

### 4.1. Dissipativity

By taking the partial derivative of the right side of Equation (9) described in the HNN, the dissipation degree of this neural network can be obtained as the following:

(10) $$\nabla V=\frac{\dot{\partial x_{1}}}{\partial x_{1}}+\frac{\dot{\partial x_{2}}}{\partial x_{2}}+\frac{\dot{\partial x_{3}}}{\partial x_{3}}+\frac{\dot{\partial z}}{\partial z}=-3+1.5{\sec h}^{2}\left(x_{1}\right)+1.5{\sec h}^{2}\left(x_{2}\right)+{\sec h}^{2}\left(x_{3}\right)-\sin\left(z\right)$$

In the equation, $\sec h\left(\cdot \right)$ is the hyperbolic secant function. Since $0\leq \sec h\left(\cdot \right)\leq 1$ and ${\sec h}^{2}\left(\cdot \right)$ is smaller, there must exist a set of solutions $\left(x_{1},x_{2},x_{3},z\right)$ such that ∇V<0. At this point, the neural network is dissipative.

### 4.2. Equilibrium Point and Its Stability

Let the right side of Equation (9) equal to 0, we can obtain the equilibrium point state equation of this neural network:

(11) $$\left\{\begin{array}{l} -x_{1}+1.5\tanh\left(x_{1}\right)+2.1\tanh\left(x_{2}\right)+\tanh\left(x_{3}\right)=0\\ -x_{2}-1.4\tanh\left(x_{1}\right)+1.5\tanh\left(x_{2}\right)=0\\ -x_{3}+2.8\tanh\left(x_{1}\right)-b\tanh\left(\left|z\right|-3\right)\tanh\left(x_{2}\right)+\tanh\left(x_{3}\right)=0\\ \cos\left(z\right)+\tanh\left(x_{2}\right)=0 \end{array}\right.$$

Solving the equilibrium point state Equation (11), we can obtain the equilibrium point of this system as follows:

(12) $$M=\{\left(\bar{x_{1}},\bar{x_{2}},\bar{x_{3}},\bar{z}\right)|\;\bar{x_{1}}=\bar{x_{2}}=\bar{x_{3}}=0,\bar{z}=\frac{1}{2}k\pi \}$$

Among them, *k* takes odd values. Find the Jacobi matrix of the system at the equilibrium point:

(13) $$J=\left[\begin{array}{cccc} 0.5 & 2.1 & 1 & 0\\ -1.4 & 0.5 & 0 & 0\\ 2.8 & -b\tanh\left(\left|\frac{k\pi }{2}\right|-3\right) & 0 & 0\\ 0 & 1 & 0 & -\sin\left(\frac{k\pi }{2}\right) \end{array}\right]$$

Let b=−4, and *k* take odd integers within the range of [−5,5]. Determine the eigenvalues and stability of the system at the equilibrium point *M* as shown in Table 1. As shown in Table 1, this system is unstable. Therefore, this neural network can generate chaotic attractors.

### 4.3. Bifurcation Diagram and Lyapunov Exponent Spectrum

**Figure 6:**
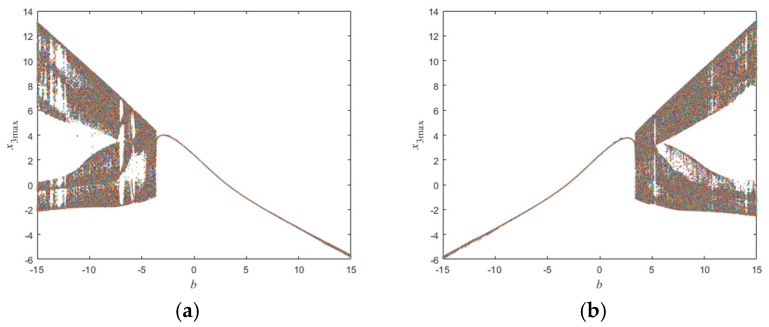
The bifurcation diagram of the synaptic coupling strength value b in the range of (−15,15), and the initial conditions of the system are (**a**) (1,1,1,3) and (**b**) (1,1,1,−3).

Set the initial conditions of this neural network ($x_{1}\left(0\right),x_{2}\left(0\right),x_{3}\left(0\right),z\left(0\right)$) to (1,1,1,3) and (1,1,1,−3), and perform numerical simulations on this neural network to obtain the bifurcation diagrams of the synaptic coupling strength value *b* in the range (−15,15), as shown in Figure 6a,b. From Figure 6, it can be seen that the dynamic behavior of the memristor HNN is influenced by the synaptic coupling strength value *b*. Comparing Figure 6a,b, it can be observed that the dynamic behavior of the memristor HNN is closely related to the initial conditions. The chaotic phenomenon implies that the long-term behavior of the system is highly sensitive, and even slight differences in initial conditions can lead to completely different outcomes.

**Figure 7:**
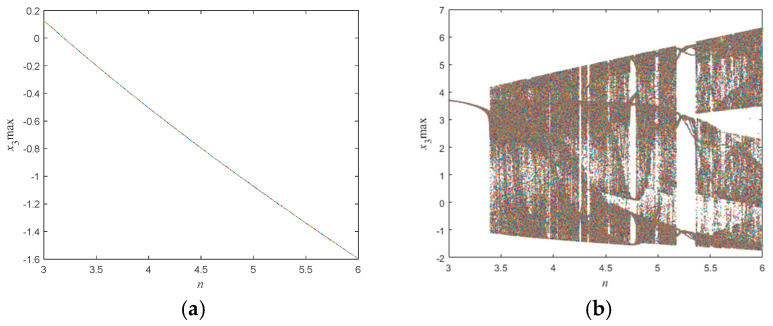
Bifurcation diagram regarding the synaptic coupling strength value *b*, the initial conditions of the system are (**a**) (1,1,1,3) and (**b**) (1,1,1,−3).

Next, to explore the detailed aspects of the dynamical behavior of this neural network, the initial conditions of the memristor HNN are set to (1,1,1,3) and (1,1,1,−3). Numerical simulations are conducted, and the bifurcation diagrams of the system with respect to the synaptic coupling strength value *b* in the range of (3,6) are shown in Figure 7a,b. From Figure 7a, it can be seen that when the initial conditions are (1,1,1,3), the system only exhibits periodic oscillation behavior within the range of b∈(3,6); when the initial conditions are (1,1,1,−3), as the system’s memristor synaptic coupling strength value *b* increases, the system transitions from the initial periodic oscillation to a complex chaotic state at b=3.40. After a period of chaotic oscillation, the system exhibits period 6 behavior within the range of $b\in \left(4.74,\;4.78\right)$. Following a brief chaotic oscillation, the system exhibits period 3 behavior within the range of $b\in \left(5.19,\;5.35\right)$. In chaotic systems, stable periodic orbits and chaotic behavior can occur simultaneously. Period 6 represents a critical phenomenon before chaos, indicating that the system’s dynamics are becoming increasingly complex; period 3 is a special case, and its appearance signifies that the system is no longer simply predictable but has entered a chaotic state.

**Figure 8:**
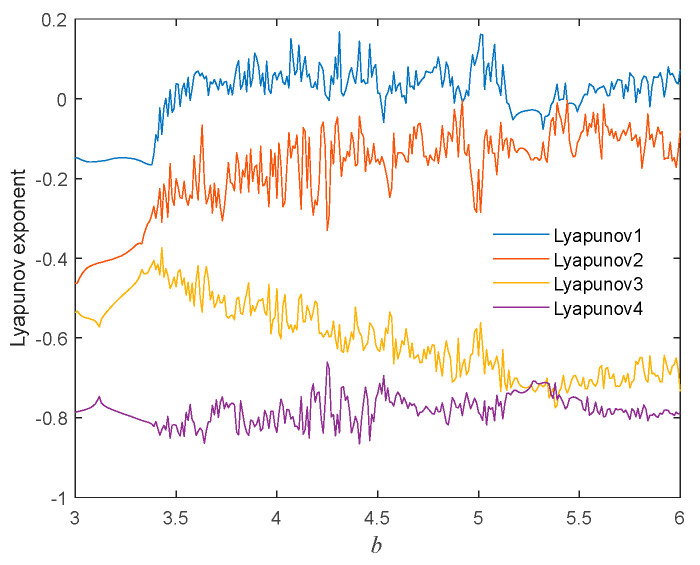
Lyapunov exponent spectrum regarding the value of synaptic coupling strength *b*.

To verify the correctness of the above analysis, the initial conditions of the system were set to (1,1,1,−3), and numerical simulations were conducted to obtain the Lyapunov exponent spectrum of the system with respect to the synaptic coupling strength value *b* in the range of (3,6), as shown in Figure 8. It is known that the Lyapunov exponent spectrum is bounded by 0; values below 0 indicate that the system is periodic at that time, while values above 0 indicate that the system is chaotic at that time. From Figure 8, it can be seen that the intervals where the Lyapunov exponent is below 0 are (3.00,3.40), (4.74,4.78), and (5.19,5.35); the intervals where the Lyapunov exponent is above 0 are (3.41,4.73), (4.79,5.18), and (5.36,6.00). Comparing the bifurcation diagram in Figure 7b with the Lyapunov exponent spectrum in Figure 8, it can be seen that the range of the parameter *b* when chaos occurs is basically consistent, thereby confirming the correctness of the above analysis.

### 4.4. Two-Dimensional Phase Trajectory Plot

**Figure 9:**
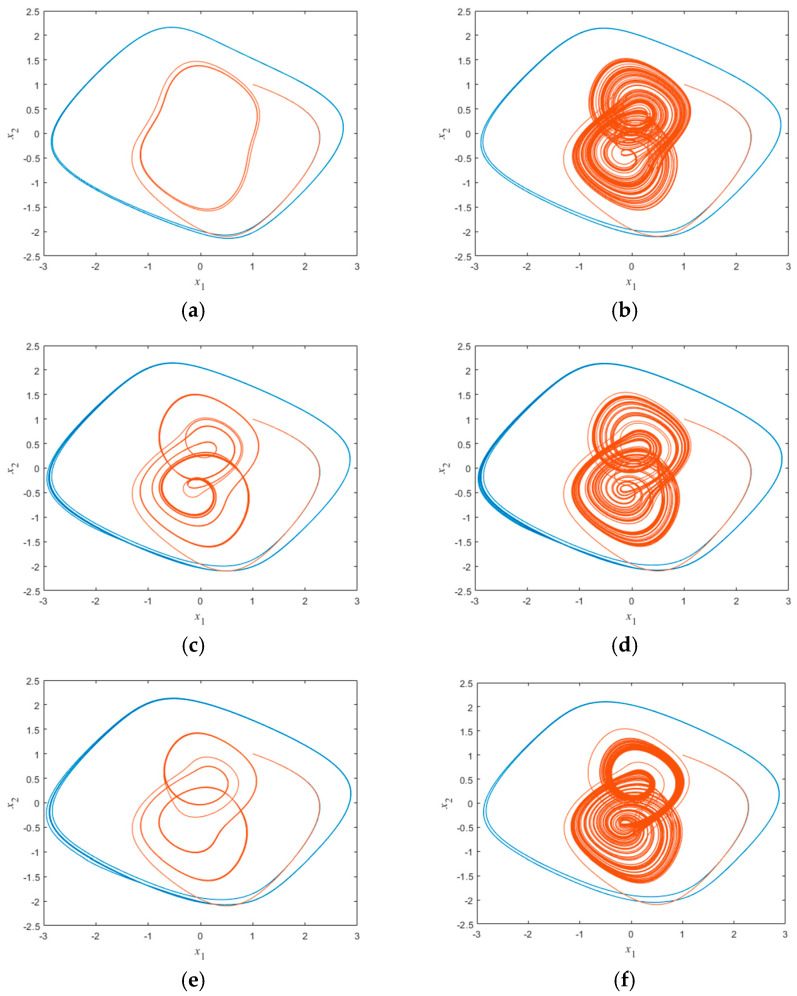
Two-dimensional phase trajectory plot for different *b* values: (**a**) coexisting period 1 attractors (b=3.00); (**b**) coexisting double scroll attractors and periodic attractors (b=4.50); (**c**) coexisting period 6 attractors period 1 attractors (b=4.74); (**d**) coexisting double scroll attractors and periodic attractors (b=5.10); (**e**) coexisting period 3 attractors period 1 attractors (b=5.24); and (**f**) coexisting double scroll attractors and periodic attractors (b=5.80).

To further illustrate the relationship between the dynamical behavior of this neural network and the memristor synapse coupling strength value *b* as well as the initial conditions, we take the initial conditions as (1,1,1,3) and (1,1,1,−3). The four-dimensional system represented by Equation (9) is numerically simulated using the fourth-order Runge–Kutta discretization algorithm, and the two-dimensional phase trajectory diagrams for different *b* values are shown in Figure 9. In the figure, the blue trajectory represents the system generated under the initial condition (1,1,1,3), while the orange trajectory represents the system generated under the initial condition (1,1,1,−3). As shown in Figure 9, when the initial condition is (1,1,1,3), the system exhibits periodic behavior with changes in the *b* value; when the initial conditions are (1,1,1,−3), the system exhibits changes from period 1 to chaos to period 6 to chaos to period 3 to chaos as the value of *b* varies.

### 4.5. Super-Multistability Phenomenon

**Figure 10:**
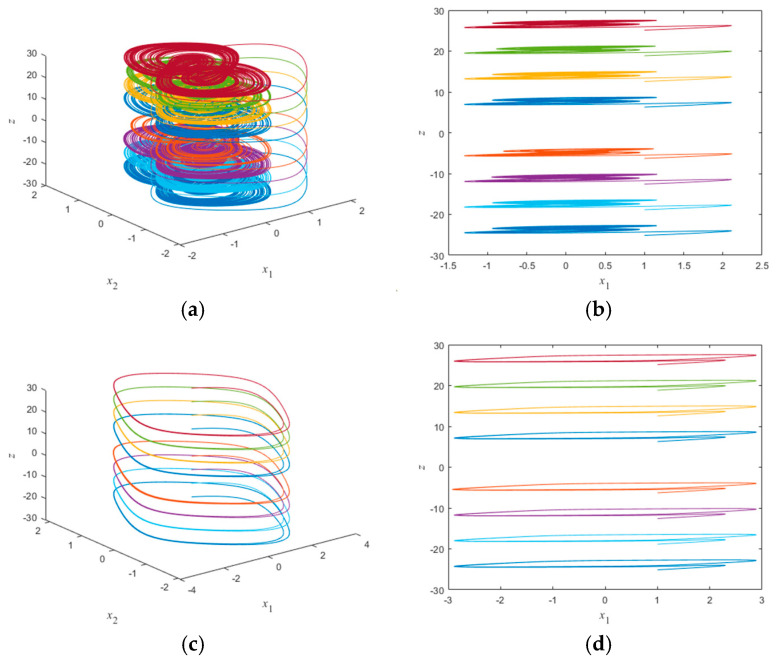
Initial conditions *z*(0) and associated coexisting chaotic and periodic attractors: (**a**) coexisting chaotic attractor in the $x_{1}-x_{2}-z$ plane; (**b**) coexisting chaotic attractor in the $x_{1}-z$ plane; (**c**) coexisting periodic attractor in the $x_{1}-x_{2}-z$ plane; and (**d**) coexisting periodic attractor in the $x_{1}-z$ plane.

When the memristor synaptic coupling strength value b=5 is set, the initial conditions $x_{1}\left(0\right)=x_{2}\left(0\right)=x_{3}\left(0\right)=1$ and *z*(0) are set to ±2π, ±4π, ±6π, and ±8π, respectively, the memristor HNN can produce the phenomenon of coexistence of 8 chaotic attractors. The three-dimensional chaotic attractors and two-dimensional chaotic attractors are shown in Figure 10a,b, respectively. When the memristor synaptic coupling strength value is set to b=−5, the initial conditions $x_{1}\left(0\right)=x_{2}\left(0\right)=x_{3}\left(0\right)=1$ and *z*(0) are set to ±2π, ±4π, ±6π, and ±8π, respectively, the memristor HNN can produce a phenomenon of coexistence of 8 periodic attractors. The three-dimensional periodic attractor and the two-dimensional periodic attractor are shown in Figure 10c,d, respectively. As shown in Figure 10, when different initial values *z*(0) are taken, the neural network can produce an infinite number of coexisting chaotic attractors and periodic attractors [30]. This system exhibits high sensitivity to the initial values of the variables. Therefore, the memristor Hopfield neural network proposed in this paper exhibits multiple steady-state phenomena, and its dynamic behavior is more complex.

### 4.6. Comparison of Different Memristor-Coupled Hopfield Neural Networks

The proposed system is compared with other systems, and the summary is shown in Table 2. From Table 2, it can be seen that there are relatively few examples of local active memristor-coupled HNNs and using digital circuits to implement in hardware. In addition to generate coexistence phenomena, this system proposed in this paper also produces super-multistability phenomenon that are not present in the following systems. This indicates that the introduction of hyperbolic tangent function leads to more complex nonlinear phenomena in the system.

## 5. FPGA Hardware Implementation

**Figure 11:**
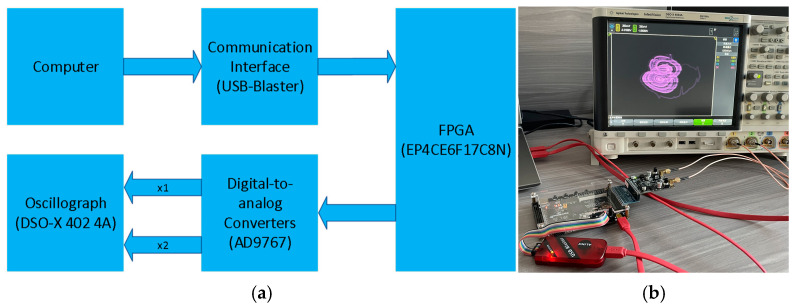
Hardware realization connection diagram of the system: (**a**) connecting block diagram of the system and (**b**) physical connection diagram of the system.

Currently, hardware implementation is primarily realized in the form of analog circuits, digital circuits, and mixed-signal circuits [31,32,33]. However, the disadvantage of analog circuits compared to digital circuits is that many electronic components in analog circuits are affected by factors such as temperature, which can reduce the accuracy of hardware implementation. Therefore, many scholars choose to use digital circuits for hardware implementation, such as, Ref. [20], Ref. [34], and Ref. [35]. This section introduces a method for implementing digital circuits based on the FPGA platform, with the hardware block diagram shown in Figure 11a. As shown in Figure 11a, the connection of this system involves first connecting the computer to the FPGA development board with the chip model EP4CE6F17C8 via the USB-Blaster communication interface and then connecting the external AD9767 digital-to-analog converter to the oscilloscope through the development board to implement the neural network in hardware.

This system first uses the Gaussian discretization algorithm to discretize the neural network. The digital iteration results are then converted into analog signals through a digital-to-analog converter. These analog signals are connected to an oscilloscope to observe whether they match the numerical simulation results. The hardware implementation based on the FPGA platform is shown in Figure 11b.

In this hardware implementation, we also encountered some practical challenges and provided optimized techniques. For example, the hardware resources of an FPGA are limited, while neural networks typically require a large amount of computational and storage resources. This makes resource allocation and optimization key issues when implementing large-scale neural networks on FPGA. We can make full use of specific resources that can significantly improve performance and resource utilization efficiency. Since FPGA hardware typically uses fixed-point arithmetic instead of floating-point arithmetic, it is necessary to carefully manage precision loss and numerical stability issues. To address the computational overhead of nonlinear functions (such as the hyperbolic tangent function), we implemented function approximation based on Lookup Tables to accelerate computation speed while maintaining accuracy.

**Figure 12:**
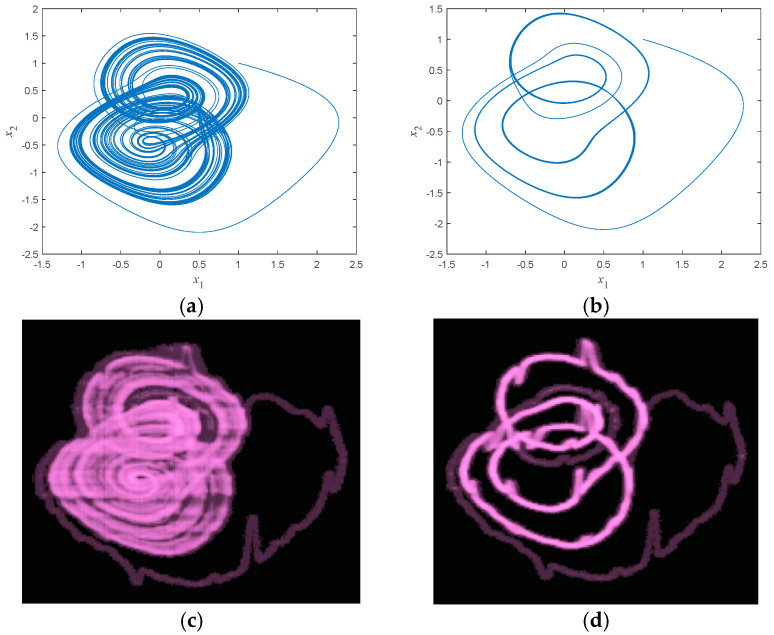
MATLAB simulation diagram and phase diagram displayed by the oscilloscope: (**a**) simulation diagram at b=5.10 (chaos); (**b**) simulation diagram at b=5.24 (period-3); (**c**) phase diagram displayed by the oscilloscope at b=5.10 (chaos); and (**d**) Phase diagram of the oscilloscope display at b=5.24 (period-3).

When the initial values are (1,1,1,−3), two specific synaptic coupling strength values *b* of 5.10 and 5.24 were selected from Section 4.4, resulting in chaotic and periodic-3 states of the system, respectively. For these two synaptic coupling strength values, the two-dimensional phase trajectory diagrams displayed on the oscilloscope and the two-dimensional phase trajectory diagrams simulated in MATLAB R2023a are basically consistent, verifying the hardware feasibility of this system. The results are shown in Figure 12a–d.

## 6. Conclusions

This paper designs an absolute value hyperbolic tangent-type dual local active memristor model, using it as a synaptic weight in a three-neuron HNN to construct a Hopfield neural network model with memristor-coupled three neurons. Through basic theoretical analysis and numerical simulation methods, the study investigated the impact of memristor synaptic coupling strength and the system’s initial conditions on the dynamics of neural networks. Finally, the hardware implementation of this neural network was carried out using an FPGA development board, verifying that the memristor-coupled three-neuron HNN constructed in this paper can be applied to practical engineering and provided optimization techniques to address practical challenges in this hardware implementation.

## Figures and Tables

**Table 1:** The eigenvalue and stability at the equilibrium point *M* when b=−4.

*b*	*k*	Eigenvalue	Stability
−4	−5	1; 1.2874 ± 1.6695i;−1.5748	Unstable saddle point
	−3	−1; 1.2711 ± 1.6415i;−1.5421	Unstable saddle point
	−1	1;−0.4222 ± 1.3302i; 1.8445	Unstable saddle point
	1	−1;−0.4222 ± 1.3302i; 1.8445	Unstable saddle point
	3	1; 1.2711 ± 1.6415i;−1.5421	Unstable saddle point
	5	−1; 1.2874 ± 1.6695i;−1.5748	Unstable saddle point

**Table 2:** Comparison of different memristor-coupled Hopfield neural networks.

System	The Type of G(*x*)	Local Active Memristor	Super-Multistability Phenomenon	Digital Circuit
Ref. [11]	linear function	no	no	no
Ref. [12]	absolute value function	no	no	no
Ref. [13]	linear function	no	no	no
Ref. [19]	quadratic function	yes	no	no
Ref. [20]	compound exponential function	yes	no	yes
This paper	hyperbolic tangent function	yes	yes	yes

## Data Availability

Data are contained within the article.
